# The Impact of Individual Depressive Symptoms on Impairment of Psychosocial Functioning

**DOI:** 10.1371/journal.pone.0090311

**Published:** 2014-02-28

**Authors:** Eiko I. Fried, Randolph M. Nesse

**Affiliations:** 1 Cluster of Excellence “Languages of Emotion”, Freie Universität Berlin, Berlin, Germany; 2 Department of Education and Psychology, Freie Universität Berlin, Berlin, Germany; 3 School of Life Sciences, Arizona State University, Tempe, Arizona, United States of America; West China Hospital of Sichuan University, China

## Abstract

Previous studies have established that scores on Major Depressive Disorder scales are correlated with measures of impairment of psychosocial functioning. It remains unclear, however, whether individual depressive symptoms vary in their effect on impairment, and if so, what the magnitude of these differences might be. We analyzed data from 3,703 depressed outpatients in the first treatment stage of the Sequenced Treatment Alternatives to Relieve Depression (STAR*D) study. Participants reported on the severity of 14 depressive symptoms, and stated to what degree their depression impaired psychosocial functioning (in general, and in the five domains work, home management, social activities, private activities, and close relationships). We tested whether symptoms differed in their associations with impairment, estimated unique shared variances of each symptom with impairment to assess the degree of difference, and examined whether symptoms had variable impacts across impairment domains. Our results show that symptoms varied substantially in their associations with impairment, and contributed to the total explained variance in a range from 0.7% (hypersomnia) to 20.9% (sad mood). Furthermore, symptoms had significantly different impacts on the five impairment domains. Overall, sad mood and concentration problems had the highest unique associations with impairment and were among the most debilitating symptoms in all five domains. Our findings are in line with a growing chorus of voices suggesting that symptom sum-scores obfuscate relevant differences between depressed patients and that substantial rewards will come from close attention to individual depression symptoms.

## Introduction

About 60% of individuals who meet criteria for Major Depressive Disorder (MDD) as defined by the Diagnostic and Statistical Manual of Mental Disorders (DSM-5) [1] report severe or very severe impairment of functioning [2]. Impairment associated with depression is long-lasting [3] and equal or greater than impairment caused by other common, chronic medical conditions such as diabetes, hypertension, heart attack, and congestive heart failure [4], [5]. Moreover, depression impairs functioning in various domains such as home life, workplace, friends, and family [6], [7] – severely compromising the capacity for self-care and independent living in many cases.

A recent review found moderate correlations between scores on various screening instruments for depression and measures of impairment [8]. It has been unclear, however, whether certain symptoms are more impairing than others, and if so, what the magnitude of these differences might be. This question is highly relevant because of large differences in the symptoms experienced by patients diagnosed with MDD.

Qualifying for a diagnosis of MDD requires experiencing at least five of the nine DSM symptomatic criteria, among which at least one has to be either sad mood or loss of interest, for at least 2 weeks. Four symptoms are compound symptoms comprised by different subsymptoms (feelings of worthlessness *or* inappropriate guilt) or opposite subsymptoms (insomnia *or* hypersomnia, psychomotor agitation *or* retardation, weight loss *or* weight gain), leading to 1,497 unique symptom profiles that all qualify for the same diagnosis [9], including profiles that do not have a single symptom in common. Considerable symptom variability has been reported across individuals [10]–[12] and within individuals across time [13], [14].

Specific depressive symptoms have received comparably little attention because they are assumed to be diagnostically interchangeable indicators of a common diagnosis. This assumption of symptom equivalence [15] goes hand in hand with the conceptualization of depression within the framework of reflective latent variable modeling [16], [17]: variation in the latent disorder depression *causes* variation of the observable symptoms. Depression is viewed as the common cause for diverse symptoms such as insomnia, psychomotor agitation, or loss of interest – which is the reason why symptoms are measured in order to assess depression. Since all symptoms indicate the same latent disease, only the *number* of symptoms is relevant, not their *natures*. The notion that different symptoms are diagnostically equivalent justifies the common practice of summing the number of symptoms to reflect depression severity.

However, several authors have suggested that there are substantial benefits to analyzing depressive symptoms individually [15], [18]–[20]. This is supported by evidence showing that symptoms differ from each other in their associations with demographic variables, personality traits, lifetime comorbidities, and risk factors [15], [21], and it has been established that specific stressful life events are predictive of distinct MDD symptom profiles [22]–[25]. Furthermore, particular gene polymorphisms are associated with specific depressive symptoms [26], [27], and a recent study of 7,500 twins concluded that the DSM symptomatic criteria for depression do not reflect a single underlying genetic factor [28].

We are aware of only a single previous study that explored concurrent effects of individual depressive symptoms on impairment of psychosocial functioning [29]. In this analysis of a general population sample, six DSM-III [30] symptoms were significantly associated with impairment (depressed mood, dysthymia, cognitive difficulties, suicidal ideation, fatigue, and sexual disinterest).

The present study extends the previous report [29] in four important aspects: (1) we examine the differential impact of symptoms on impairment in a large and highly representative sample of 3,703 depressed patients; (2) we use the updated DSM-5 criterion symptoms; (3) we investigate subsymptoms (e.g., psychomotor agitation and psychomotor retardation) instead of compound symptoms (e.g., psychomotor problems); (4) lastly, we test whether symptoms vary in their impacts across five impairment domains.

## Materials and Methods

### Study description

Data from the first treatment stage (level 1) of the NIH-supported “Sequenced Treatment Alternatives to Relieve Depression” (STAR*D) study [31], [32] were analyzed for this report. Data can be obtained from the NIMH and were provided to the authors under terms of an NIHM Data Use Certificate that protects confidentiality; dataset version 3 was used. STAR*D was a multisite randomized clinical trial conducted in the USA to investigate which of several treatment options would be most effective for nonpsychotic MDD outpatients; 4,041 patients were enrolled into the first treatment stage, in which all participants received citalopram, a selective serotonin reuptake inhibitor (SSRI) antidepressant. Outcome data were obtained via telephone interviews that were conducted either by interviewers, or by an interactive voice response system (IVR). STAR*D was approved and monitored by the institutional review boards at each of the 14 participating institutions, a national coordinating center, a data coordinating center, and the data safety and monitoring board at the NIMH. All participants provided written informed consent at study entry. Detailed information about design, methods, exclusion criteria, and the rationale of STAR*D are described elsewhere [31], [32].

### Participants

STAR*D used relatively inclusive selection criteria in order to obtain a highly representative sample of patients seeking treatment for MDD. Participants had to be between 18 and 75 years, fulfill DSM-IV criteria for single or recurrent nonpsychotic MDD, and have at least moderately severe depression corresponding to a score of at least 14 on the 17-item Hamilton Rating Scale for Depression (HAM-D) [33]. Participants with a history of bipolar disorder, schizophrenia, schizoaffective disorder, or psychosis were excluded, as were patients with current anorexia, bulimia, or primary obsessive compulsive disorder. Further exclusion criteria were a history of intolerability to antidepressant medication, lack of response to an adequate trial of SSRI in the current episode of MDD, or failure to respond to 16 or more sessions of cognitive therapy in the current episode of MDD. Our analyses are limited to the 3,703 individuals that were assessed within the first week of level 1 via IVR.

### Outcomes measures

STAR*D used the Quick Inventory of Depressive Symptoms (QIDS-16 [34]) to assess depressive symptoms. The QIDS-16 has good psychometric properties [34], and the results of the IVR version are comparable to the results produced by the self-rated and the clinician-rated QIDS-16 [35]. The QIDS-16 assesses the nine DSM symptom domains with 16 questions (Table 1). Each domain yields a score between 0 and 3, 0 indicating no problems, 3 indicating severe problems. While six symptoms are measured with single questions, the three compound symptoms (*sleep problems*, *psychomotor problems*, *appetite*/*weight problems*) are assessed with multiple questions. The QIDS-16 constructs these compound symptoms by using the highest symptom score in each symptom group, resulting in one score on each of the nine DSM criterion symptoms. Since we were interested in individual symptoms, we used all available items instead of symptom domains. Detailed information for the domain *appetite and weight problems* was not available, since either *appetite decrease* or *appetite increase*, and either *weight decrease* or *weight increase* was scored. Overall, this resulted in twelve individual symptoms plus the two compound symptoms *appetite problems* and *weight problems* (Table 1).

**Table 1:** Depressive symptoms.

QIDS-16 symptoms	Shortcode
Sleep onset insomnia	Early insomnia
Mid-nocturnal insomnia	Middle insomnia
Early morning insomnia	Late insomnia
Hypersomnia	Hypersomnia
Sad Mood	Sad mood
Appetite increase	Appetite
Appetite decrease	Appetite
Weight increase	Weight
Weight decrease	Weight
Problems concentrating/making decisions	Concentration
Feeling worthless/self-blame	Self-blame
Suicidal ideation	Suicidal ideation
Loss of interest	Interest loss
Energy loss/fatigability	Fatigue
Psychomotor slowing	Slowed
Psychomotor agitation	Agitated

The Work and Social Adjustment Scale (WSAS [36]) was used to measure impairment of functioning. The WSAS is a simple, reliable, and valid self-report instrument that uses Likert-scale ratings of 5 items to assess impairment in the domains of work, home management, social activities, private activities, and close relationships. Each question is rated on a 0–8 Likert scale, with 0 indicating no impairment and 8 indicating very severe impairment. WSAS scores below 10 are associated with subclinical populations; scores of 10–20 are associated with significant functional impairment, while scores above 20 suggest at least moderately severe functional impairment (total range 0–40). The WSAS has been used mainly in samples with mood and anxiety disorders, and has been shown to have good internal consistency (0.70 to 0.94) and retest-reliability (0.73), and high concurrent validity of IVR administrations with clinician interviews (0.81 and 0.86) [37]. In STAR*D, the WSAS specifically queried participants how much *their depression* impaired work and social activities. For instance, work impairment was measured via the following item: “Because of my depression, my ability to work is impaired. 0 means not at all impaired and 8 means very severely impaired to the point I can't work.”

### Statistical analysis

Three analyses were performed. First, we used the 14 QIDS-16 depression symptoms to predict overall impairment as measured by the WSAS sum-score, controlling for age and sex. We then compared two linear regression models: in model I (heterogeneity model), regression weights for symptoms were free to vary, whereas model II (homogeneity model) constrained regression weights to be equal. While model I allows for differential impairment-symptoms associations, model II represents the hypothesis that symptoms have equal associations with impairment. A *χ* ^2^-test was used to compare the two models. Because depressive symptoms are generally correlated with each other, we performed multicollinearity diagnostics for both regression analyses. The variance inflation factor (VIF) did not exceed the value of five for any symptom, indicating no multicollinearity problems [38].

Second, we aimed to allocate unique *R* ^2^ shares (proportion of explained variance) to each regressor to examine how much unique variance each individual symptom shared with impairment. We used the LMG metric via the R-package RELAIMPO [39] to estimate the relative importance (RI [40]–[42]) of each symptom. LMG estimates the importance of each regressor by splitting the total *R* ^2^ into one non-negative *R* ^2^ share per regressor, all of which sum to the total explained *R* ^2^. This is done by calculating the contribution of each predictor at all possible points of entry into the model, and taking the average of those contributions. In other words, an estimate of RI for each variable is obtained by calculating as many regressions as there are possible orders of regressors (in the present case, 8.7×10^10^ regressions), and then averaging individual *R* ^2^ values over all models. RI estimates are then adjusted to sum to 100% for easier interpretation. Confidence interval (CI) estimates of the RI coefficients, as well as *p*-values indicating whether regressors differed significantly from each other in their RI contributions (in an exploratory sense), were obtained using the bootstrapping capabilities of the RELAIMPO package. It is important to note that predictors with a non-significant regression coefficient can nonetheless contribute to the total explained variance, that is, have a non-zero LMG contribution. This is the case when regressors are correlated with each other and thus can indirectly influence the outcome via other regressors [42]. Therefore, all symptoms, even those without significant regression coefficients, were included in subsequent RI calculations.

Third, we tested whether individual symptoms differed in their associations across the five WSAS impairment domains work, home management, social activities, private activities and close relationships. We estimated two structural equation models (SEM), using the Maximum-Likelihood Estimator. Both models contained five linear regressions, one for each domain of impairment. In each of these five regressions, we used the 14 depressive symptoms as predictors of one impairment domain, controlling for age and sex. While the first SEM allowed free estimation of all regression coefficients (model I), the second constrained each symptom to have equal effects (i.e. regression coefficients) across the five impairment domains (model II). This second model represents the hypothesis that a given symptom has similar impacts on all five domains. We compared the models using a *χ* ^2^-test.

Analyses one and three were performed in MPLUS v7.0 [43], and analysis two was estimated in R v2.13.0 [44].

## Results

Of the 3,703 outpatients in the study, 2,234 (60.3%) were female, and the mean age was 41.2 years (*sd* = 13.2). See Table 2 for detailed demographic information.

**Table 2:** Demographic characteristics.

Category	Subcategory	Subjects (%)
Age	≤20 y	86 (2.3)
	21–30 y	842 (22.7)
	31–40 y	835 (22.5)
	41–50 y	915 (24.7)
	51–60 y	711 (19.2)
	>60 y	314 (8.5)
Race	White	2926 (79.0)
	Black or African American	685 (18.5)
	Other	92 (2.5)
Ethnicity	Hispanic	452 (12.2)
Marital Status	Never married	1091 (29.5)
	Cohabitating with partner	310 (8.4)
	Married	1238 (33.4)
	Separated	245 (6.6)
	Divorced	698 (18.8)
	Widowed	117 (3.2)
	Missing	4 (0.1)
Employment status	Unemployed	1379 (37.3)
	Employed	2101 (56.8)
	Retired	218 (5.9)
	Missing	5 (0.1)

The average impairment score was 23.52 (*sd* = 9.29), corresponding to moderately severe levels of impairment; 307 (8.3%) individuals did not show impaired functioning, 875 (23.6%) exhibited significant functional impairment, while 2,521 (68.1%) reported severe functional impairment.

### Homogeneity versus heterogeneity of associations

The heterogeneity model (allowing variable contributions of symptoms to impairment) fit the data significantly better than the homogeneity model (in which symptoms were constrained to have the same contributions to impairment) (*χ* ^2^ = 394.5, *df* = 13, *p*<0.001). In the heterogeneity model, 11 of the 14 depression symptoms as well as male sex and older age significantly predicted impairment, explaining 40.8% of the variance (*F* (16, 3686) = 159.1, *p*<0.001) (Table 3). The heterogeneity model was thus used for subsequent RI estimations.

**Table 3:** Results of linear regression analysis (heterogeneity model).

Predictors	*b*	*s.e.*	*t*	
Early insomnia	0.50	0.11	4.53	***
Middle insomnia	0.01	0.15	0.08	
Late insomnia	0.26	0.11	2.32	*
Hypersomnia	0.54	0.15	3.64	***
Sad mood	2.27	0.18	12.79	***
Appetite	0.25	0.12	2.14	*
Weight	0.13	0.11	1.17	
Concentration	1.61	0.14	11.21	***
Self-blame	0.68	0.10	6.61	***
Suicidal ideation	0.84	0.15	5.50	***
Interest loss	1.24	0.12	10.40	***
Fatigue	1.08	0.12	8.78	***
Slowed	0.84	0.14	5.93	***
Agitated	0.02	0.13	0.13	
Age	0.04	0.01	4.07	***
Sex	−0.31	0.25	−1.25	

*b*, unstandardized regression coefficient; *s.e.*, standard error; *t*, *t*-value;

* *p*<0.05;

** *p*<0.01;

*** *p*<0.001.

### Relative importance analysis

**Figure 1:**
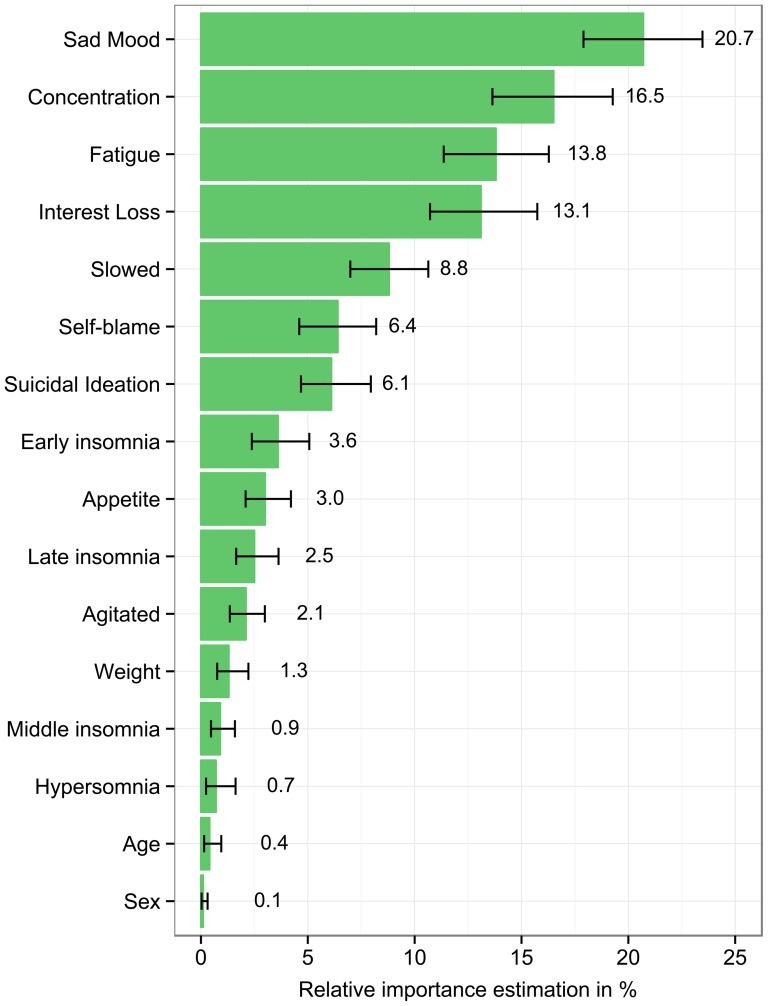
Relative importance coefficients of depressive symptoms on overall impairment. Relative importance coefficients of depressive symptoms on overall impairment, including bootstrapped confidence intervals. Each value represents the unique shared variance between a symptom and impairment, controlling for age and sex. Estimates are adjusted to sum to 100%.

The RI estimates of all regressors, representing the allocated individual R^2^ contributions of symptoms on impairment, are displayed in Figure 1. Different symptoms had drastically different effects on impairment, ranging from RI values of 0.7% (*hypersomnia*) to 20.9% (*sad mood*). Out of 91 symptom pairs, 76 (83.5%) significantly differed in their RI contributions to impairment (all *p*<0.05). RI coefficients within the two compound symptoms (*sleep problems* and *psychomotor problems*) showed differential RI: *early insomnia* (3.6%) was associated with significantly more impairment than *middle insomnia* (0.8%) and *hypersomnia* (0.7%), while *slowed* (8.7%) had a significantly larger RI estimate than *agitated* (2.1%) (all *p*<0.05).

Are the large differences in the impact of different symptom on disability due to the *nature* of symptoms, or due to their *severity*? If severity, then severity differences between symptoms should explain a large proportion of the differences of the RI estimates (i.e. symptoms with high mean values are highly debilitating, whereas symptoms with a low mean are associated with much less impairment). To test this hypothesis we used a linear regression to predict the RI of each of the 14 symptoms by its mean severity. Symptom severity did not reach statistical significance as predictor for symptom RI estimates (*F* (1,12) = 4.0, *p* = 0.07). This implies that RI differences are due to symptom nature, and not symptom severity.

### Impact of symptoms across impairment domains

**Figure 2:**
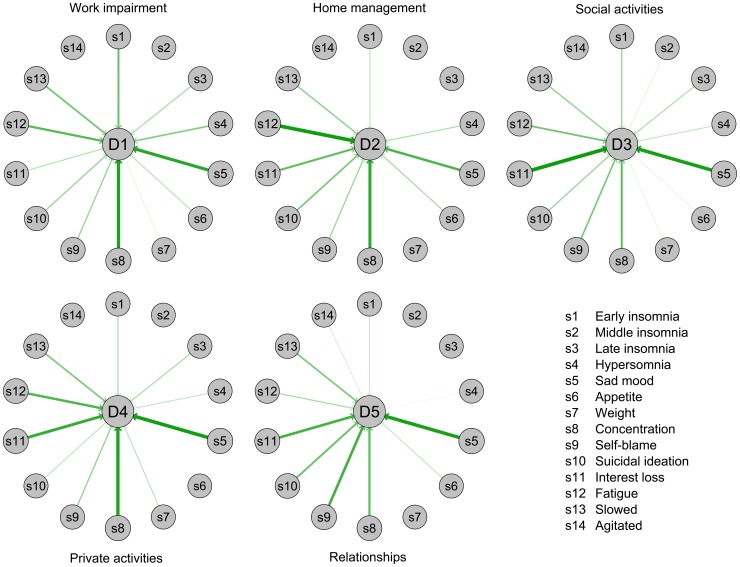
Associations between depressive symptoms and impairment domains. The arrows represent standardized regression coefficients of the 14 QIDS-16 depression symptoms (s1–s14) on the five WSAS impairment domains (D1–D5). Thickness of arrows indicates strength of regression weights.

Constraining regression weights of symptoms to be equal across the five domains of impairment in model II significantly reduced model fit compared to model I in which symptom contributions were freely estimated (*χ* ^2^ = 299.8, *df* = 56, *p*<0.001). This means that symptoms have differential impacts across impairment domains; these differences between the symptoms-impairment associations across domains are visualized in Figure 2. Of the diverse findings, three are especially noteworthy:

(1) *sad mood* and *concentration* were among the four most debilitating symptoms in all domains;

(2) *early insomnia* had comparably strong effects on work impairment, *self-blame* on close relationships, *interest loss* on social activities, and *fatigue* on home management;

(3) compared to other domains, *interest loss* was less impairing for the domain work, *fatigue* for close relationships, *sad mood* for home management, and *concentration* for social activities as well as close relationships.

## Discussion

Overall, individual depressive symptoms have differential effects on impairment, confirming our main hypothesis. Depressed mood, poor concentration, fatigue and loss of interest explained a large proportion of variance in impairment, whereas weight problems, mid-nocturnal insomnia and hypersomnia made few unique contributions to impairment.

Subsymptoms within symptom domains had differential effects as well. For instance, psychomotor retardation explained roughly four times as much variance of impairment as psychomotor agitation. These findings highlight not only the importance of considering the nine DSM symptoms individually, but also the importance of considering sub-symptoms within the symptom domains. The three most debilitating symptoms include one affective, one cognitive and one somatic symptom, suggesting the need to monitor all kinds of depressive symptoms instead of focusing on only one domain or factor score. Furthermore, the two DSM MDD core symptoms, depressed mood and interest loss, made high contributions to explaining impairment, ranking 1 (20.7%) and 4 (13.1%) in general RI estimates. Lastly, although some symptoms were roughly equally debilitating across different domains of impairment, the majority of symptoms varied in their influence across domains.

### Implications

While prior research has established that symptoms are differentially associated with demographic variables and personality traits [15], risk factors [21], stressful life events [22]–[25], and gene polymorphisms [26]–[28], our report reveals yet another dimension of covert heterogeneity: symptoms have variable associations with impairment of psychosocial functioning. The broad depression diagnosis not only obscures important differences between patients and lumps individuals suffering from diverse symptoms into the same category – two patients with the same number of depressive symptoms may differ drastically in their functioning levels. This concealed variability within MDD potentially explains some of the most prominent “disappointing” findings portrayed in recent literature: (1) the DSM-V field trials [45] reported a “questionable” inter-rater reliability of 0.28 (CI 0.20–0.35) for MDD diagnosis, lower than the majority of other disorders (e.g., borderline personality disorder 0.54 (CI 0.43–0.66)); (2) antidepressants are only marginally efficacious compared to placebos, in spite of substantial publication and reporting bias inflating apparent antidepressant efficacy [46]; (3) there are few consistencies between studies investigating which brain regions are involved in the pathophysiology of MDD [47]; (4) none of more than half a million common genetic markers were associated with antidepressant response in a study with 1,790 individuals [48]; (5) lastly, no single locus reached genome-wide significance in a genome-wide association study of 17 population-based samples containing 34,549 subjects [49].

The dependent variable in all studies is either a symptom sum-score, or the categorical distinction between depressed and healthy. In both cases, potentially important information about symptoms is lost, and a closer examination of these symptoms is likely to reveal important insights hidden by analyses of sum-scores. In the present study, sleep onset insomnia had comparably strong impact on functioning in the domain of work. It has also been established that MDD treatment is less effective in patients suffering from sleep problems [50], that patients with persistent sleep problems are more than twice as likely to remain depressed [51], and that targeting sleep problems in patients diagnosed with MDD increases overall depression improvement [52], [53]. This example elucidates how clinically useful symptom-based approaches can be: they provide detailed information about the nature of problems individuals suffer from, and thus offer the opportunity to improving MDD prevention and treatment.

In addition to studying individual MDD criterion symptoms of depression, it is important to acknowledge that the current DSM symptoms are but a small subset of possible depression symptoms, and were determined largely by clinical consensus instead of empirical evidence [15], [54]. Several non-DSM MDD symptoms merit closer examination and should be assessed in future studies of depressive symptoms, because they are highly prevalent and associated with worse clinical outcomes. For example, studies found anxiety and anger/irritability to be present in more than half of the patients diagnosed with MDD [55], [56], and while remission of MDD was less likely and took longer in patients reporting anxiety [56], anger/irritability was a clinical marker of a more severe, chronic, and complex depressive illness [55].

### Limitations

The results have to be interpreted in the light of five limitations. First, although the impairment scale used in the STAR*D study specifically instructed participants to rate the effects of their depression on functioning, both depressive symptoms and functional impairment were assessed at the same measurement point, so caution about causal interpretations is warranted. Symptoms and impairment potentially reinforce each other and are thus likely to blur, especially in individuals suffering from chronic depression. Second, while subjects at baseline of STAR*D were not taking antidepressant medication, many participants reported other medical conditions for which prescribed medications might have affected symptom reports. Third, the bootstrapped CIs for the RI estimates are fairly large for a sample of 3,703 subjects, implying a moderate amount of model uncertainty due to the high number of regressors as well as substantial covariation between them. Fourth, item wording may have biased the associations of individual symptoms with impairment; in particular, because subjects were asked to rate the impact of their depression on impairment, sadness may be artificially inflated. To explore this further would require alternative question wording. Lastly, differential variability in depressive symptoms is a potential source of biased RI estimates, because heavily skewed symptoms with means close to the minimum and maximum are less likely to demonstrate pronounced statistical relationships. However, symptom means that ranged from 0.44 (insomnia) to 2.35 (mid-nocturnal insomnia) did not significantly predict RI estimates, and even the symptom with the lowest mean of 0.44 (insomnia) showed substantial variability (*sd* = 0.83; *sd* range of all other symptoms excluding insomnia: 0.83 to 1.21).
